# Is REDD1 a Metabolic *Éminence Grise*?

**DOI:** 10.1016/j.tem.2016.08.005

**Published:** 2016-12

**Authors:** Christopher Lipina, Harinder S Hundal

**Affiliations:** 1 Division of Cell Signalling and Immunology, Sir James Black Centre, School of Life Sciences, University of Dundee, Dundee, DD1 5EH, UK

**Keywords:** REDD1, skeletal muscle, insulin, protein kinase B, Akt, mTOR, obesity

## Abstract

Regulated in development and DNA damage response 1 (REDD1) has been functionally linked to the control of diverse cellular processes due, at least in part, to its ability to repress mammalian or mechanistic Target of Rapamycin (mTOR) Complex-1 (mTORC1), a key protein complex controlled by hormonal and nutrient cues. Notably, emerging evidence suggests that REDD1 also regulates several pathways involved in modulating energy balance and metabolism. Herein, we discuss evidence implicating REDD1 as a key modulator of insulin action and metabolic function, including its potential contribution to mitochondrial biology and pancreatic islet function. Collectively, the available evidence suggests that REDD1 has a more prominent role in energy homeostasis than was previously thought, and implicates REDD1 as a potential therapeutic target for treatment of metabolic disorders.

## Trends

REDD1 has been implicated in a range of cellular processes due, at least in part, to its ability to interact with proteins that impact protein homeostasis and metabolic function

Several obesity-related factors, including hyperinsulinaemia, hyperlipidaemia, and hypoxia, have been shown to upregulate REDD1, which may facilitate the development of insulin resistance through impaired PKB/Akt-directed signalling.

Genetic inhibition of REDD1 has been reported to impair insulin sensitivity through dysregulation of the mTORC1/S6K pathway.

Emerging evidence suggests that REDD1 acts as a key regulator of mitochondrial oxidative capacity and lipogenesis, as well as modulating insulin production and secretion.

Understanding the role of REDD1 in insulin action and energy homeostasis may provide a roadmap toward designing strategies to counteract obesity-related metabolic disorders.

## Introduction

Type 2 diabetes mellitus (T2DM) is an increasingly common metabolic disorder characterised by insulin resistance and hyperglycaemia, resulting in dysregulated uptake and storage of glucose and other metabolic substrates in peripheral tissues, such as liver, adipose, and skeletal muscle [1]. Importantly, the peptide hormone insulin acts to promote an overall hypoglycaemic response by stimulating the uptake of glucose into peripheral tissues, such as skeletal muscle and adipose tissue (in contrast to hepatocytes, which largely depend upon non-insulin-dependent facilitated diffusion of glucose via the GLUT2 glucose transporter), as well as promoting its conversion into storage molecules, such as glycogen and fat. Upon binding to its receptor, insulin triggers a series of signalling events that stimulate the translocation of the glucose transporter, GLUT4, to the cell membrane. Central to this process is the signalling adaptor molecule insulin receptor substrate 1 (IRS-1), which facilitates the activation of PI3K and downstream PKB/Akt-dependent signalling [1]. Notably, PKB/Akt functions as a key signalling node that facilitates many of the metabolic actions of insulin, including the uptake of glucose and its subsequent conversion into glycogen, through its ability to phosphorylate and inhibit the Rab GTPase-activating protein AS160 and glycogen synthase kinase 3 (GSK3), respectively 2, 3, 4. Consequently, alterations to components of insulin-induced signalling, including impairment of PKB/Akt, are likely to contribute to the pathogenesis of insulin resistance as well as perturbed glucose and lipid homeostasis.

**Figure 1:**
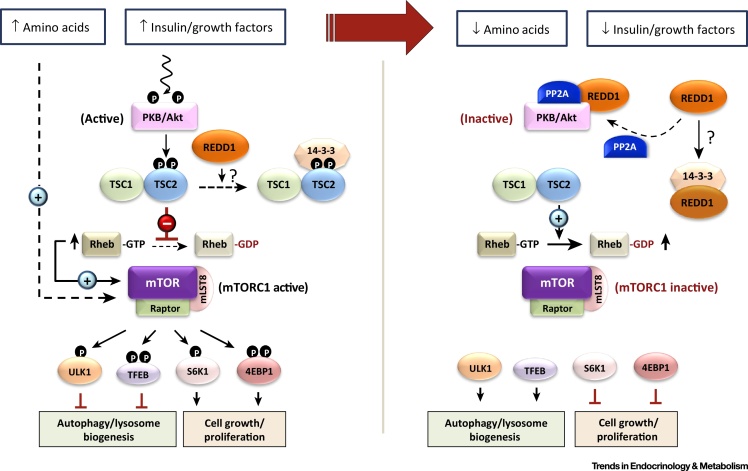
Factors Implicated in Insulin/Growth Factor-Induced Regulation of Mammalian or Mechanistic Target of Rapamycin (mTOR) Complex-1 (mTORC1). In response to cellular stimulation by mitogens (e.g., insulin/growth factors), activation of the mTORC1 protein complex leads to phosphorylation of several downstream targets, such as Unc-51-like autophagy-activating kinase 1 (ULK1), transcription factor EB (TFEB), S6K1, and 4EBP1, to regulate processes such as autophagy, protein synthesis, and cell growth and proliferation. The presence of insulin/growth factors leads to increased protein kinase B (PKB/Akt) activity, which, in turn, phosphorylates and inhibits the upstream repressor of mTORC1, known as tuberous sclerosis complex 2 (TSC2, which forms a complex with TSC1). This consequentially reduces the GTPase-activating protein (GAP) activity of TSC2 towards the G-protein Rheb, which then accumulates in its GTP-bound active form, thereby promoting stimulation of its target mTORC1. By contrast, following insulin/growth factor deprivation, diminished PKB/Akt activity leads to increased TSC2 GAP activity towards Rheb and reduced mTORC1 activation. Evidence also implicates a role for regulated in development and DNA damage response 1 (REDD1) in modulating the TSC2-Rheb-mTORC1 pathway, whereby REDD1 may act to sequester 14-3-3 proteins away from TSC2, enabling the latter to then repress Rheb activity. Alternatively, REDD1 has been suggested to facilitate the association of protein phosphatase 2A (PP2A) with PKB/Akt, leading to inactivation of the protein kinase, which, in turn, would promote increased TSC2 GAP activity towards Rheb and subsequent repression of mTORC1. In addition to mitogenic stimuli, amino acids also confer a potent stimulatory effect on mTORC1 (Box 2 and Figure 2, main text). Black arrows indicate a stimulatory effect, whereas blunt red arrows indicate an inhibitory action.

**Figure 2:**
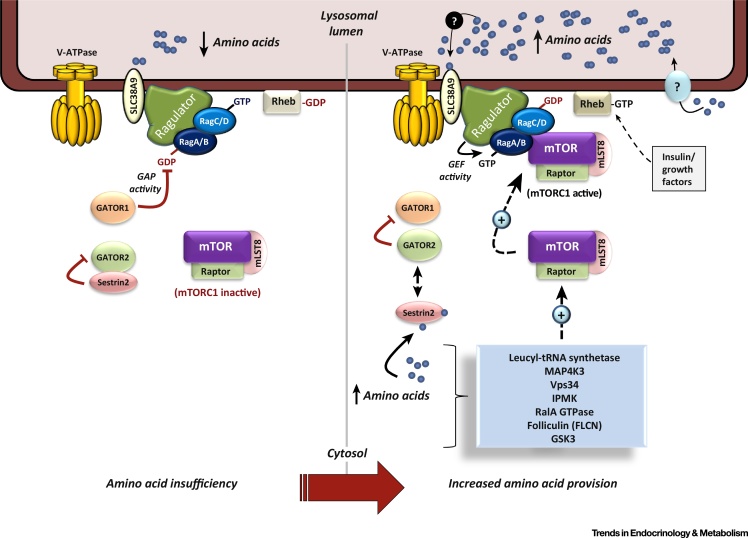
Pathways Implicated in Amino Acid Modulation of Mammalian or Mechanistic Target of Rapamycin (mTOR) Complex-1 (mTORC1). Schematic illustrates the involvement of the Rag proteins, and the Ragulator and GATOR Complexes in the lysosomal targeting and activation of mTORC1 following amino acid provision (see Box 2 for details). In addition, other proposed factors implicated in the amino acid regulation of mTORC1 are also included (see Box 2 for overview). Abbreviation: GEF, guanine nucleotide exchange factor.

REDD1, also known as DDIT4 or RTP801, has been identified as a key stress-regulated protein whose expression becomes elevated in response to a variety of cellular stressors, including hypoxia, cellular iron depletion, and DNA damage 5, 6, 7, 8. Several studies have identified REDD1 as a potent inhibitor of mTORC1, a complex whose core proteins include the serine/threonine protein kinase mTOR, mLST8, and the rapamycin-sensitive scaffolding protein Raptor. This protein complex can further associate with its inhibitory proteins DEP domain-containing mTOR-interacting protein (DEPTOR) and proline-rich Akt substrate 40 (PRAS40). mTORC1 is activated in response to mitogens (insulin and growth factors) and nutrients (amino acids) and serves to coordinate the effects of such stimuli to regulate diverse cellular processes, including protein synthesis, autophagy, and cell growth (Figure 1, Figure 2, Box 1, Box 2) 6, 9, 10. Current thinking dictates that this inhibitory action is mediated through the ability of REDD1 to activate the upstream repressor of mTORC1, known as the tuberous sclerosis complex 1 (TSC1, hamartin)/tuberous sclerosis complex 2 (TSC2, tuberin) complex [6]. One mechanism by which REDD1 may convey its repressive action towards mTORC1 is through its reported ability to facilitate the protein phosphatase 2A (PP2A)-mediated dephosphorylation and inactivation of the protein kinase PKB/Akt, which functions as an upstream repressor of TSC2 (Box 1 and Figure 1) [11]. Alternatively, it has been suggested that REDD1 acts to sequester 14-3-3 proteins away from TSC2, thereby allowing TSC2 to repress mTORC1 function (Box 1 and Figure 1) [12], although it should be highlighted that REDD1 and 14-3-3 proteins may not physically interact as described by previous structure-based docking and functional analyses [13]. Growing evidence suggests that REDD1 has an important role in modulating the activities of Akt and mTOR, two protein kinases that function to regulate cell growth and metabolism 6, 7, 11, 14, 15. In this review, we explore the evidence pertaining to the involvement of REDD1 in cellular pathways and processes that are known to impact insulin sensitivity and energy homeostasis.

## Regulation of Insulin Sensitivity by REDD1

Increasing evidence from several independent studies suggests that REDD1 functions as a key modulator of insulin action. For example, a study by Dungan and colleagues first demonstrated that mice deficient for REDD1 exhibited impaired glucose and insulin tolerance compared with wild-type counterparts [16]. This coincided with a reduction in the insulin-stimulated phosphorylation of PKB/Akt at its key regulatory residues Thr308/Ser473, as well as reduced IRS-1 tyrosine phosphorylation in skeletal muscle, indicative of reduced insulin sensitivity [16]. Consistent with this, an independent study by Regazzetti and coworkers also reported impaired insulin sensitivity in REDD1-silenced 3T3-L1 adipocytes, as evidenced by reduced phosphorylation of PKB/Akt, as well as muted tyrosine phosphorylation of the insulin receptor and IRS-1 in response to insulin [17]. Indeed, these findings suggest that reduced functionality of proximal insulin receptor signalling underlies the impairment in insulin action conveyed by a loss in REDD1 function. In accord with this, the study by Regazzetti and colleagues also demonstrated the restoration of insulin signalling by the mTORC1 inhibitor rapamycin, thereby suggesting that the impaired insulin action observed in response to REDD1 deficiency is dependent upon mTOR activity. The mTORC1 complex is known to activate negative feedback pathways that function to downregulate IRS-1 function by promoting its serine phosphorylation 18, 19. Therefore, it is possible that impaired insulin action observed in response to REDD1 silencing may arise due to an aberrant increase in mTORC1/S6K-dependent negative-feedback control [20]. Several independent groups have reported dysregulation of mTOR signalling, as evidenced by hyperactive S6K1 phosphorylation within skeletal muscle of obese *ob/ob* and diet-induced mice 15, 21, 22, 23, 24. In agreement with these findings, Regazzati and coworkers further revealed the ability of mTOR inhibition to enhance insulin-induced tyrosine phosphorylation of the insulin receptor in REDD1-silenced 3T3-L1 adipocytes [17]. Furthermore, metformin, an insulin-sensitising drug, has been reported to inhibit mTOR as well upregulate REDD1 expression in prostate cancer cells [25]. However, whether this biguanide acts to improve insulin action through elevating REDD1 expression and/or activity remains unknown.

Therefore, the prevailing paradigm would suggest that tissue expression of REDD1 would be lower in the obese and/or diabetic state. However, several studies have reported elevated REDD1 protein expression in skeletal muscle in various animal models of obesity and/or diabetes, including obese *ob/ob* and high-fat diet (HFD)-fed mice, as well as streptozotocin-induced diabetic mice 14, 15, 26, 27, 28. Although it remains unclear whether elevated REDD1 expression contributes to repressed PKB/Akt-directed signalling *in vivo*, work by Dennis and colleagues recently demonstrated that REDD1 acts to enhance PP2A-mediated dephosphorylation of PKB/Akt at Thr308 [11]. Moreover, REDD1 was also shown to co-immunoprecipitate with PKB/Akt and the catalytic subunit of PP2A, suggesting that REDD1 acts to bridge the interaction between PKB/Akt and the phosphatase under certain cellular conditions [11]. In line with this, the authors of the same study demonstrated that REDD1 mediated the interaction of PKB/Akt with PP2A in response to endoplasmic reticulum (ER) stress. Interestingly, REDD1 was only found to enhance PP2A-mediated dephosphorylation of PKB/Akt at the Thr308 residue in its catalytic domain, and not at the Ser 473 regulatory site in its hydrophobic motif [11]. This suggests that REDD1 acts to modulate site-specific activity of PP2A, although how it does so remains unclear. It is noteworthy that recent work by Moore and coworkers demonstrated the ability of glucosamine to repress insulin-stimulated PKB/Akt Thr308 phosphorylation and downstream mTORC1 signalling in retinal Müller cells by increasing ER stress and REDD1 protein expression [29]. However, the potential involvement of PP2A in mediating the insulin-desensitising action of glucosamine was not determined in this study.

In agreement with these findings, insulin-induced phosphorylation of PKB/Akt Thr308 was shown to be augmented in REDD1-deficient mouse embryonic fibroblasts (MEFs), concomitant with enhanced insulin-stimulated phosphorylation of the two PKB/Akt substrates GSK3α/β (Ser21/9) and Foxo1/3a (Thr24/32) [11]. It is noteworthy that this contrasts with observations made in REDD1-deficient mice and REDD1-silenced 3T3-L1 adipocytes, in which reduced insulin sensitivity has been reported 16, 17. Therefore, this suggests that the modulatory effects of REDD1 upon insulin action are cell type and context specific, particularly in response to the genetic manipulation of REDD1 by gene silencing and/or its ectopic expression, and will require further investigation. Indeed, based on the previously reported observation that active PP2A can mediate palmitate-induced insulin resistance [30], it is plausible that the ability of the saturated fatty acid (or lipid intermediates derived from it, such as ceramide) to inhibit PKB/Akt may be mediated, at least in part, through promoting REDD1-dependent interactions between PP2A and PKB/Akt. To this end, further work will be required to establish the relative contribution of REDD1 in mediating the insulin-desensitising actions of saturated fatty acids, such as palmitate. Moreover, the development of strategies aimed at reducing REDD1 expression and/or activity, such as through increased physical exercise 31, 32, 33, may prove beneficial in counteracting the deleterious metabolic effects associated with the obese and/or insulin-resistant state.

In addition, obesity is often associated with the state of hyperinsulinaemia 34, 35. Notably, insulin itself has been shown to transiently induce REDD1 expression in human and murine adipocytes through activation of the PI3K/mTOR pathway and hypoxia-inducible factor-1 (HIF1), a transcriptional activator of the REDD1 gene [36]. Moreover, a recent study by Williamson and coworkers reported increased REDD1 protein expression in skeletal muscle of individuals with T2DM following a hyperinsulinaemic-euglycaemic clamp [37]. Interestingly, however, no such increase was observed in response to insulin in corresponding lean control counterparts. Accordingly, insulin-stimulated mTOR activation, as determined by the difference between basal and insulin-induced S6K1 phosphorylation, was found to be significantly higher in insulin-treated lean individuals, whereas it remained unchanged in the T2DM group [37]. In accord with these findings, prolonged insulin exposure has been shown to attenuate the insulin-signalling capacity by promoting inhibitory serine phosphorylation of IRS-1 through activation of serine/threonine kinases, such as JNK, IKK, S6K1, and mTOR 18, 38, 39. Therefore, from a physiological perspective, the ability of insulin to upregulate REDD1 expression in skeletal muscle could be envisioned as a regulatory loop to restore basal signalling, and/or as a contributing factor in the development of insulin resistance.

Together, these findings indicate that REDD1 may act to impair insulin action under conditions where its expression and/or activity becomes either aberrantly elevated, for example in obesity and/or diabetes, or when it is substantially suppressed (i.e., through genetic inhibition). This suggests that tightly coordinated regulation of REDD1 expression and/or activity is crucial for maintaining proper insulin action in peripheral tissues, such as skeletal muscle.

## Potential Factors Implicated in the Modulation of REDD1 Expression

Emerging evidence suggests that tissue REDD1 protein expression becomes altered in response to obesity [15], thereby implicating several obesity-related factors in its regulation. For example, increased adiposity has been associated with ER stress in various peripheral tissues, including adipose tissue and skeletal muscle [40]. Moreover, ER stress has been shown to induce *REDD1* gene expression through activation of its transcriptional regulator ATF4 41, 42. However, whether ER stress promotes insulin resistance, either *in vitro* or *in vivo*, through the induction of REDD1 remains unclear.

Alternatively, another obesity-related factor that may regulate REDD1 expression is the hypoxia-regulated transcription factor HIF1α [36], which has also been implicated in the development of insulin resistance. Overexpression of HIF1α in adipose tissue has been reported to induce insulin resistance and glucose intolerance [43]. Conversely, adipocyte-specific disruption of HIF1α ameliorated HFD-induced insulin resistance in mice [44]. In obesity, adipose tissue undergoes expansion in response to excess caloric intake, eventually becoming hypoxic due to the inability of the vasculature to keep pace with tissue growth. Consequently, the resulting hypoxic conditions act to upregulate HIF1α levels in adipose tissue 45, 46, 47. However, it remains unclear whether REDD1 mediates the insulin-desensitising actions of HIF1α in the obese state. To address this, future work may, for example, involve exploring the metabolic effects of targeted REDD1 silencing in adipose tissue of transgenic mice expressing HIF1α.

Obesity-induced alterations in lipid levels may also act to modulate REDD1 expression. Work by Williamson and colleagues suggests that elevated circulating and/or tissue lipid levels increase skeletal muscle REDD1 expression 15, 48. For example, mice fed a HFD exhibit elevated REDD1 protein abundance in skeletal muscle 15, 48, 49. Moreover, this diet-induced increase in muscle REDD1 expression was shown to be attenuated following calorie restriction in mice [49]. Elevated levels of REDD1 protein have been reported in ceramide-treated C2C12 myotubes, concomitant with reduced Akt phosphorylation [48]. Allied to these findings, recent work by Lee and colleagues demonstrated that lipopolysaccharide (LPS), a proinflammatory lipid known to act by targeting Toll-like receptors, such as TLR2 and TLR4, induces REDD1 expression in macrophages via a CREB-dependent mechanism [50]. Saturated fatty acids, such as palmitate, have also been reported to bind and activate TLR2 and TLR4 [51]; however, it remains to be determined whether saturated (e.g., palmitate; C16:0) and/or unsaturated (e.g., oleate; C18:1) fatty acids directly regulate REDD1 expression. Further work will be required to establish the role of REDD1 in mediating fatty acid-induced insulin resistance and metabolic dysfunction, for example by utilising REDD1-deficient cells (i.e., myotubes, hepatocytes, and/or adipocytes).

REDD1 expression can also be altered in response to changes in nutritional status. For example, food deprivation is known to induce an increase in circulating glucocorticoids, such as corticosterone, which in turn acts to stimulate REDD1 expression through the glucocorticoid receptor (GR) 14, 52. Given that glucocorticoids are able to induce insulin resistance, it is plausible that their insulin-desensitising effects may be mediated, at least in part, through increased REDD1 [53]. In accord with this, circulating glucocorticoid (corticosterone) levels have been shown to be elevated in type 2 (*db/db* mice) diabetic mice, concomitant with increased muscle REDD1 expression [26]. Moreover, co-treatment of *db/db* mice with a GR antagonist was found to normalise REDD1 muscle expression without altering serum glucocorticoid concentrations [26]. However, the effects of the GR antagonist upon insulin sensitivity were not described in this study. Further evidence supporting a role for REDD1 in mediating the biological actions of glucocorticoids was revealed by work demonstrating protection against the atrophic effects of topical glucocorticoid application in skin cells of REDD1-knockout mice [54], as well as the prevention of dexamethasone-induced skeletal muscle atrophy in REDD1-null mice [55]. Alternatively, elevated REDD1 mRNA abundance in white adipose tissue, liver, and skeletal muscle of fasted mice has been suggested to be induced by the activation of p53, as evidenced by the ability of the p53 activator nutlin-3 to increase REDD1 mRNA abundance in C3H10T1/2 adipocytes [56]. Therefore, changes in the activity of distinct factors, such as glucocorticoid levels and p53 activity, may underlie altered REDD1 expression in different nutritional states, with further analysis required to establish how these may impact insulin action and other metabolic parameters.

## REDD1-Mediated Regulation of Mitochondrial Function

In accord with its reported participation in the modulation of insulin sensitivity, there is now emerging evidence to suggest that REDD1 also regulates mitochondrial integrity and oxidative capacity. A study by Horak and coworkers first demonstrated that a significant portion (>10%) of REDD1 is localised to mitochondria in MEFs [57]. The authors of the same study also showed that this localisation was required for REDD1 to suppress mitochondrial reactive oxygen species (ROS) production. Specifically, mitochondrial preparations from REDD1-deficient MEFs exhibited increased generation of multiple ROS species, including superoxide (^**.**^O2–) and peroxide (H_2_O_2_), compared with corresponding controls [57]. Strikingly, retroviral reconstitution of REDD1 was found to normalise cellular ROS production in REDD1-deficient MEFs. By contrast, expression of a mutant form of REDD1 with compromised mitochondrial localisation failed to reduce cellular ROS levels in the same manner [57]. In agreement with these findings, the antioxidant salidroside was reported to prevent hydrogen peroxide-induced apoptosis of human umbilical vein endothelial cells (HUVECs) in a REDD1-dependent manner, which coincided with the attenuation of excessive ROS generation, as well as the ability of the antioxidant to upregulate REDD1 protein levels [58]. However, it should be stressed that REDD1 may also act to positively modulate intracellular ROS production, as demonstrated by elevated ROS levels in response to REDD1 ectopic expression in TP63-null fibroblasts, as well as reduced hydrogen peroxide content in splenocytes of REDD1-deficient mice 59, 60. In the latter case, reduced oxidative activity following a loss in REDD1 expression may be linked to mitochondrial dysfunction, as evidenced by a recent study reporting decreased basal oxygen consumption, oxidative ATP generation, and maximal respiratory capacity in REDD1-deficient MEFs [60]. The same study also reported that reduced autophagic flux induced by treadmill exercise in skeletal muscle of REDD1-deficient mice coincided with the accumulation of defective mitochondria, leading to impaired oxidative phosphorylation [60]. In mechanistic terms, REDD1 modulation of autophagy has been suggested to involve its direct interaction with the pro-oxidant protein TXNIP, whereby genetic suppression of either REDD1 or TXNIP in MEFs has been shown to reduce cellular ROS and increase the catalytic activity of the redox-sensitive ATG4B cysteine endopeptidase, leading to increased LC3B delipidation and impaired autophagy [60]. Indeed, because mitochondrial dysfunction has been linked with the development of insulin resistance 61, 62, 63, 64, it is conceivable that decreased mitochondrial oxidative capacity may, at least in part, contribute toward the impaired insulin action observed in response to REDD1 deficiency. Consistent with this, REDD1-deficient mice were shown to display a marked reduction in exercise capacity compared with wild-type counterparts, concomitant with significantly reduced (∼30%) ATP levels in skeletal muscle following a forced exercise regime [60]. Separate studies also revealed the ability of resistance exercise to induce rapid but transient increases in REDD1 expression (mRNA and protein) in gastrocnemius muscle of rats 65, 66, as well as in the vastus lateralis muscle of older male individuals [67]. However, the exact mechanisms underlying these exercise-induced increases in REDD1 expression, and whether they are linked to improvements in mitochondrial oxidative capacity and insulin sensitivity, as well as changes in redox homeostasis and autophagic capacity, remain unknown.

In addition to the production of ROS, other mechanisms may also be involved in REDD1-mediated regulation of mitochondrial function. For example, PKB/Akt can promote cell survival in part by blocking apoptosis initiated in response to cytochrome c release from mitochondria 68, 69. Moreover, reduced PKB/Akt activity in response to altered REDD1 expression may contribute to impaired mitochondrial function by compromising the ability of PKB/Akt to preserve mitochondrial function through targeting downstream effectors, such as Pim-1 and the FOXO and Hexokinase isoforms, particularly in response to cellular stress 69, 70, 71, 72, 73.

Notably, mTORC1 has also been reported to positively regulate mitochondrial biogenesis and oxidative capacity 74, 75. For example, active mTORC1 acts to stimulate mitochondrial biogenesis and metabolism through transcriptional regulators, such as peroxisome proliferator-activated receptor gamma coactivator 1-alpha (PGC1α) and transcription factor yin-yang 1 (YY1) [76]. Therefore, increased REDD1 expression and/or activity may act to suppress mTORC1 activity, which, in turn, would impact negatively on mitochondrial abundance, integrity, and/or oxidative capacity. Consistent with this idea, a study by Lafarge and coworkers demonstrated that the livers of mice deficient for the protease cathepsin S exhibited a lower rate of hepatocellular respiration compared with control counterparts, concomitant with elevated hepatic expression of REDD1 [77]. In addition, peritoneal sepsis has been reported to increase REDD1 protein content in skeletal muscle of mice [78], as well as to promote derangements in mitochondrial bioenergetics in this tissue [79]. Therefore, further work will be required to explore the potential link between obesity and/or diabetes, REDD1, and mitochondrial function, for example using relevant animal models of obesity and/or diabetes as well as REDD1 deficiency.

## REDD1 Modulation of Insulin Production and Secretion

Although little is known regarding the role of REDD1 in the regulation of pancreatic function, a study by Williamson and coworkers revealed that circulating plasma insulin concentrations were significantly lower in mice lacking REDD1 compared with wild-type counterparts, suggesting that insulin secretion and/or production become impaired in response to REDD1 deficiency [15]. Consistent with this idea and the fact that REDD1 acts to negatively regulate mTORC1, β cell failure has been shown to coincide with mTORC1 hyperactivation, an effect demonstrated in mice harbouring a β cell-specific deletion of TSC2, a key negative upstream regulator of mTORC1 [80]. Conversely, disruption of mTORC1 function by silencing the expression of raptor, a key component of the mTORC1 complex, was shown to concur with increased glucose-stimulated insulin secretion and intracellular insulin content in pancreatic INS-1 cells [81]. Therefore, the anticipated hyperactivation of mTORC1 in response to a loss of REDD1 may act to reduce insulin production and secretion, as well potentiate β cell failure, as observed in the diabetic state. Further work will be required to characterise the role of REDD1 in the regulation of insulin production and secretion, as well as β cell viability, particularly in the obese and diabetic states. To this end, it is plausible that observed reductions in insulin action in mice lacking REDD1 may, at least in part, be due to a state of insulin deficiency as well as insulin resistance [16].

## REDD1 Involvement in Lipid Metabolism

Although the involvement of REDD1 in the regulation of lipogenesis remains poorly understood, work by Regazzetti and coworkers first demonstrated that silencing REDD1 in 3T3-L1 adipocytes using siRNA resulted in the attenuation of insulin-induced lipogenesis, concomitant with suppressed insulin action [17]. Accordingly, REDD1 may be implicated in the control of lipogenic pathways through its ability to modulate insulin signalling and, in particular, the activity of downstream PKB/Akt targets, such as the transcription factor sterol regulatory element binding protein-1 (SREBP-1), which has a crucial role in lipid homeostasis by inducing the expression of genes, such as those encoding acetyl-CoA carboxylase (*ACC*) and fatty acid synthase 82, 83. In addition to REDD1-mediated effects on lipogenesis, a separate study by Schupp and coworkers also revealed that forced expression of REDD1 was sufficient to induce lipolysis in cultured C3H10T1/2 adipocytes, as evidenced by increased glycerol and free fatty acid release [56]. Interestingly, this REDD1-driven enhancement in lipolysis did not coincide with significant alterations to lipolytic genes, such as those encoding adipose triglyceride lipase (*ATGL*) and hormone sensitive lipase (*HSL*) [56]. Therefore, given the emerging evidence for REDD1 in the modulation of lipid homeostasis, further work will be required to determine those factors that can mediate the regulatory actions of REDD1 on lipolytic and lipogenic processes, particularly *in vivo*.

## Concluding Remarks and Future Perspectives

**Figure 3:**
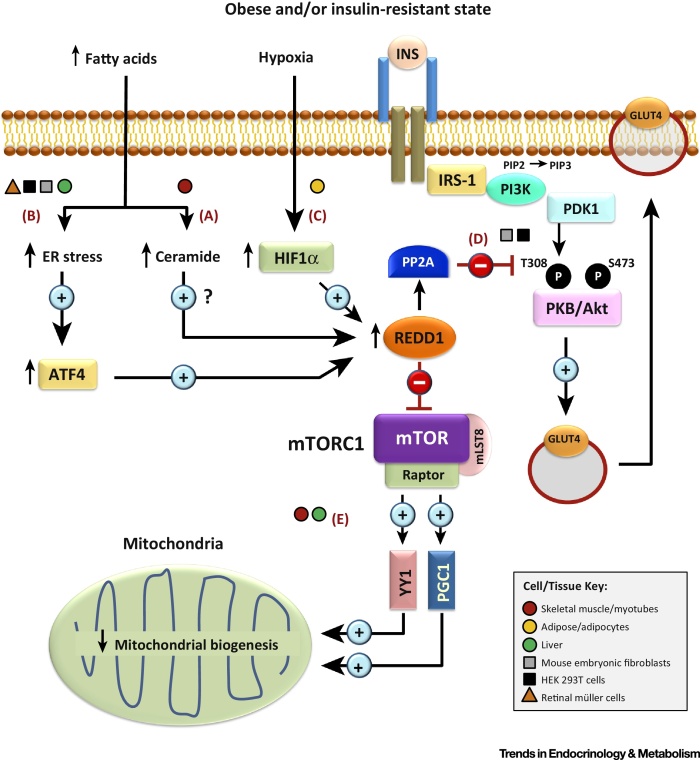
Proposed Mechanisms Involved in Regulated in Development and DNA Damage Response 1 (REDD1)-mediated Insulin Resistance and Mitochondrial Dysfunction. Obesity-related increases in circulating saturated fatty acids (FAs), such as palmitate (C16:0), promote the generation of lipid intermediates, such as ceramide (A), as well as inducing endoplasmic reticulum (ER) stress (B). In addition, obesity is associated with elevated levels of the transcription factor hypoxia-inducible factor 1 alpha (HIF1α), for example in expanding adipose tissue (C). These factors may contribute to increased REDD1 expression, at least in part, through stimulating transcriptional regulators, such as ATF-4 and HIF1α. Elevated REDD1 then acts to inhibit PKB/Akt by promoting its dephosphorylation at Thr308 by protein phosphatase 2A (PP2A), leading to the downregulation of key downstream processes, such as GLUT4-dependent glucose uptake (D). Increased REDD1 levels would also act to suppress mammalian or mechanistic Target of Rapamycin (mTOR) Complex-1 (mTORC1) activity, which, in turn, may lead to decreased mitochondrial biogenesis through attenuating gene regulation by transcriptional modulators, such as peroxisome proliferator-activated receptor gamma coactivator 1 (PGC1) and yin-yang 1 (YY1) (E). The schematic also includes the type of tissues/cells in which the signalling event has been shown to occur or be implicated, as denoted by the key. Abbreviation: IRS-1, insulin receptor substrate 1.

**Figure 4:**
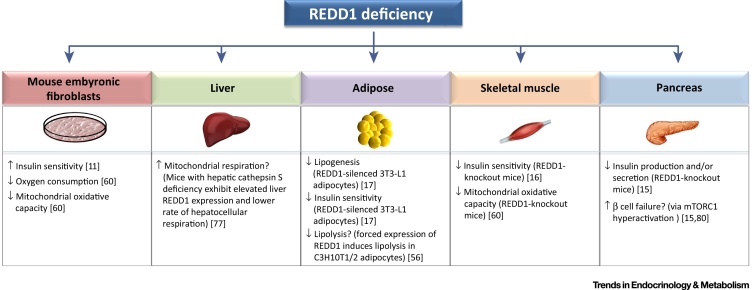
Metabolic Effects Associated with Regulated in Development and DNA Damage Response 1 (REDD1) Inhibition/Deficiency. Schematic illustration of how global REDD1 deficiency impacts insulin sensitivity and other metabolic processes in different peripheral tissues (or derived cell lines in which REDD1 has been silenced) as well as in mouse embryonic fibroblasts (MEFs). Supporting references are highlighted in square brackets. Question marks denote a process that is hypothesised (for example, based on evidence demonstrating the opposite effect in response to REDD1 overexpression). Arrows (↑/↓) indicate either an increase or decrease, respectively. Abbreviation: mTORC1, mammalian or mechanistic Target of Rapamycin (mTOR) Complex-1.

To conclude, there is growing evidence supporting a role for REDD1 in modulating insulin sensitivity and other processes that impact energy homeostasis. This may be through the ability of REDD1 to alter the function of key insulin-signalling components, as well as modulating cellular (mitochondrial) bioenergetics (Figure 3, Figure 4). Notably, we have highlighted various metabolic responses associated with REDD1 induction (Figure 3) or inhibition (Figure 4) that reveal distinct modes of action. However, several issues remain, including the involvement of REDD1 in mediating obesity-induced insulin resistance and metabolic dysfunction in different peripheral tissues (i.e., skeletal muscle, adipose tissue and liver) (see Outstanding Questions), as well as the therapeutic impact of targeting REDD1 to counteract such metabolic perturbations. Moreover, little is known regarding the role of REDD1 in modulating pancreatic islet function, and how the function of this protein may influence other obesity-related pathologies, such as the development of hepatic steatosis and/or cardiovascular disease. Given the diverse nature of potential REDD1-interacting proteins and regulators (e.g., TXNIP, PP2A, HIF1α, p53, and 14-3-3 proteins) 11, 12, 36, 56, 60, it is possible that the involvement of REDD1 in regulating energy homeostasis is multifaceted and, as outlined in this review, may impact numerous metabolic pathways and processes. Therefore, future work investigating the functional role of REDD1 may offer new insights into the pathogenesis of metabolic deficiencies associated with obesity and diabetes, as well as providing novel strategies for their prevention. In addition, the reported metabolic actions of REDD1 may also have wide-ranging implications regarding its involvement in the pathogenesis of various other diseases, such as cancer and neurological disorders, where dysregulation of cellular metabolism has a critical role in their development 12, 84.

Outstanding Questions What role does REDD1 have in the pathogenesis of obesity-induced insulin resistance? Are the metabolic effects of REDD1 induction and/or suppression cell type or tissue specific?

What strategies (pharmacological, genetic, or otherwise) can be implemented to counteract obesity-related changes in REDD1 expression and/or signalling that impact negatively insulin sensitivity and glucose homeostasis?

Does islet β cell failure in T2DM coincide with altered REDD1 expression and/or function in this tissue?

Can regulation of REDD1 expression and/or function be used as a therapeutic strategy to control ectopic fat accumulation (steatosis) in the liver and heart?

## Box 1 Key Factors Implicated in Insulin/Growth Factor Regulation of mTORC1 Activity

mTOR is an essential component of the protein complex known as mTORC1, which functions to integrate mitogenic and nutrient signals to promote the growth and proliferation of cells under nutritionally favourable conditions (i.e., in response to provision of growth factors and amino acids). This is mediated, to a large extent, by the ability of active mTORC1 to phosphorylate and regulate several downstream effectors, such as p70S6 kinase 1 (S6K1) and 4E-BP1, which have important roles in the regulation of protein synthesis (Figure 1, main text) 6, 7.

From a mechanistic viewpoint, activation of mTORC1 is crucially dependent upon a small G-protein called Rheb, which, in its GTP-loaded active form, can bind and stimulate mTORC1. The relative amounts of GTP-loaded (active) and GDP-loaded (inactive) Rheb are determined by its intrinsic GTP hydrolysing activity, which in turn is directly modulated by the GTPase**-**activating protein (GAP) activity of the tuberous sclerosis complex (TSC1/2) [85]. TSC2 is a direct substrate for the protein kinase PKB/Akt, whose stimulation by insulin and growth factors induces phosphorylation of TSC2 and subsequent inhibition of its GAP activity (Figure 1, main text) [9]. This results in the accumulation of active Rheb-GTP and an associated increase in mTORC1 activity.

In comparison, inhibition of mTORC1 may, in part, be mediated through altered localisation of the TSC1/2 complex. For example, the absence of amino acids and insulin has been shown to induce translocation of the TSC1/2 complex to the lysosomal surface, where it acts to convert Rheb into its inactive GDP-bound form 86, 87. Conversely, provision of these stimuli prevents lysosomal targeting of the TSC1/2 complex and maintains Rheb in its active (GTP-bound) state. mTORC1 can also be negatively regulated by REDD1, whose expression is increased in response to cellular stresses, such as hypoxia or iron deprivation 6, 8. Although the mechanism(s) underlying this inhibitory action are not fully understood, it has been suggested that REDD1 acts by sequestering 14-3-3 proteins away from TSC2, thereby permitting GAP activity of TSC2 towards its target Rheb (Figure 1, main text) [12]. Furthermore, recent work indicates that forced expression of REDD1 promotes the association of PP2A with PKB/Akt, leading to the dephosphorylation and inactivation of the kinase on one of its key regulatory sites (Thr308). Consequently, this would restrain PKB/Akt-mediated phosphorylation and inhibition of TSC2, resulting in the downstream inactivation of Rheb and mTORC1 [11].

## Box 2 Amino Acid Regulation of mTORC1 Signalling

While mTORC1activation by amino acids (AAs) does not directly involve TSC1/2 [88], it relies crucially upon mobilising mTORC1 from the cytosol to the lysosomal membrane surface, where sensing of AA sufficiency is thought to occur. This AA-induced translocation of mTORC1 involves members of the Rag family of small G-proteins that operate as functional heterodimers (RagA or RagB with RagC or RagD) [89] and requires RagA/B to be GTP loaded and the RagC/D component to be GDP loaded. In this configuration, Rags can interact with mTORC1 and tether to the Ragulator (Rag regulator) complex [89], thereby placing mTOR in close proximity to its activator Rheb-GTP (Figure 2) [90].

While the Ragulator has guanine nucleotide exchange factor (GEF) activity towards RagA/B to facilitate GDP exchange for GTP, hydrolysis of bound GTP is influenced by another protein complex, GATOR1, which has GAP activity towards RagA/B [91]. GATOR1 is itself regulated by another protein complex, GATOR2, whose activity, in turn, is influenced by association with a protein called Sestrin2. AA insufficiency promotes Sestrin2 association with GATOR2, which represses its action toward GATOR1, allowing the latter to hydrolyse RagA/B-bound GTP and maintain mTORC1 in the cytosol. By contrast, the Sestrin2 and GATOR2 association is reduced upon AA provision, which allows inhibition of GATOR1 and permits Rag-dependent mTORC1 translocation to lysosomes. In addition to Sestrin2, recent work has also identified a lysosomal membrane AA transporter, SLC38A9 (aka SNAT9), as a putative AA sensor whose occupancy by substrate AAs may signal AA sufficiency to mTORC1 via its interaction with the Rag-Ragulator complex and the vacuolar H^+^-ATPase 92, 93.

However, while recruitment of the mTORC1 protein ensemble to lysosomes is important for AA-induced mTOR activation, this process may be subject to additional AA sensing/regulatory inputs (Figure 2, main text). For example, leucyl tRNA synthetase, MAP4K3 (a mitogen-activated protein kinase kinase), Vps34 (a class III phosphatylinositol 3-kinase), inositol polyphosphate multikinase (IPMK), folliculin, and RalA have all been implicated in the nutrient-induced activation of mTORC1 94, 95, 96, 97, 98. More recent work has indicated that glycogen synthase kinase-3 (GSK3)-mediated phosphorylation of raptor may have a permissive role in AA-dependent mTORC1 signalling through stabilising the mTOR/raptor interaction, thus supporting phosphorylation of raptor-bound mTOR substrates [99]. Whether these additional inputs act in concert to orchestrate nutrient-dependent mTORC1 activation or whether their involvement is cell type and context specific is currently unclear.
