# Supplementary material for: Is REDD1 a Metabolic Éminence Grise?
Source: Trends Endocrinol Metab. 2016 Dec;27(12):868–80. doi: 10.1016/j.tem.2016.08.005 (PMC5119498; doi:10.1016/j.tem.2016.08.005)
Supplement: Supplementary file 1 [file mmc1.pdf]

Question 1:

Which organelles have been implicated in facilitating mTORC1 activation by amino acids?

❍ Endoplasmic reticulum

❍ Mitochondria

● Lysosomes

Explanation:

mTORC1 is recruited to lysosomes for activation in response to amino acid provision.

Reference:

Shimobayashi, M. and Hall, M.N. (2016) Multiple amino acid sensing inputs to mTORC1. Cell Res 26, 7-20

❍ Peroxisomes

Question 2:

Which regulatory sites on PKB/Akt has REDD1 been shown to facilitate the dephosphorylation of?

❍ Serine 473 only

● Threonine 308 only

Explanation:

REDD1 over expression or stress induced expression of REDD1 induces a selective loss in Akt Thr308 phosphorylation but not that of Akt Ser473

Reference:

Dennis, M.D., et al. (2014) REDD1 enhances protein phosphatase 2A-mediated dephosphorylation of Akt to repress mTORC1 signaling. Sci Signal 7, ra68; Watson, A., et al. (2016) Iron depletion suppresses mTORC1-directed signalling in intestinal Caco-2 cells via induction of REDD1. Cellular signalling 28, 412-424

❍ Both Serine 473 and Threonine 308

❍ Threonine 34 only

Question 3:

Which molecule is not a physiological substrate for PKB/Akt?

❍ AS160

● REDD1

Explanation:

AS160, GSK-3 and TSC2 are all phosphorylated by PKB/Akt, whereas REDD1 is not

Reference:

Laplante, M. and Sabatini, D.M. (2012) mTOR signaling in growth control and disease. Cell 149, 274-293; Sano, H., et al. (2003) Insulin-stimulated phosphorylation of a Rab GTPase-activating protein regulates GLUT4 translocation. J Biol Chem 278, 14599-14602

❍ Glycogen Synthase Kinase-3

❍ TSC2

Question 4:

Which of the following are most likely to be true? Note: GAP, GTPase-Activating protein; GEF, guanine nucleotide exchange factor

● REDD1 can inhibit mTORC1 by stimulating the GAP activity of TSC2

Explanation:

TSC2 possesses GAP activity towards Rheb. REDD1 suppresses Akt signalling resulting in reduced TSC2 phosphorylation and hence greater retention of GAP activity towards Rheb and thus inhibition of mTORC1.

Reference:

Shimobayashi, M. and Hall, M.N. (2016) Multiple amino acid sensing inputs to mTORC1. Cell Res 26, 7-20

❍ REDD1 can inhibit mTORC1 by inhibiting the GAP activity of TSC2

❍ REDD1 can inhibit mTORC1 by activating the GEF activity of TSC2

❍ REDD1 can inhibit mTORC1 by activating the GEF activity of TSC2

Question 5:

Which of the following statements is correct?

● REDD1 deficiency induces a decrease in cellular ROS production.

Explanation:

Genetic suppression of REDD1 in MEFs has been shown to reduce cellular ROS

Reference:

Qiao, S., et al. (2015) A REDD1/TXNIP pro-oxidant complex regulates ATG4B activity to control stress-induced autophagy and sustain exercise capacity. Nat Commun 6, 7014

❍ REDD1 over-expression enhances PKB/Akt signaling.

- ❍ REDD1 deficiency in adipocytes stimulates lipogenesis
- ❍ REDD1 deficiency stimulates mitochondrial oxidative capacity
- ❍ REDD1 deficiency stimulates pancreatic insulin secretion
